# Characteristics and prognostic implications of peripheral blood lymphocyte subsets in patients with anti-MDA5 antibody positive dermatomyositis-interstitial lung disease

**DOI:** 10.1186/s12890-023-02706-y

**Published:** 2023-10-28

**Authors:** Fang-Ping Ren, Qi Chen, Shan-Shan Yao, Lin Feng, Xin-Ying Xue, Wei-Chao Zhao, Dong Wang, Zhi-Ling Zhao, Si-Wei Gu, Ting Li, Ya-Wen Shen, Lan Gao, Xue-Lei Zang, Xin-Yu Bao, Zhao-Hui Tong

**Affiliations:** 1 Beijing Shijitan Hospital, Capital Medical University, Beijing, China; 2 University of Pittsburgh, Pittsburgh, USA; 3 Beijing Chao-Yang Hospital, Capital Medical University, Beijing, China; 4 PLA Strategic Support Force Medical Center, Beijing, China; 5 Xuanwu Hospital, Capital Medical University, Beijing, China; 6 Weifang Medical College, Weifang, China

**Keywords:** Anti-melanoma differentiation-associated gene 5, Dermatomyositis, Interstitial lung disease, Lymphocyte subsets, Prognostic

## Abstract

**Objectives:**

To examine the characteristics of blood lymphocyte subsets in dermatomyositis-interstitial lung disease (DM-ILD) inflicted patients with positive anti-melanoma differentiation-associated gene 5 (anti-MDA5), as well as its prognosis value in this set of patients.

**Methods:**

Data were retrospectively collected from 253 DM-ILD patients from three hospitals in China between January 2016 to January 2021. Patients were grouped into anti-MDA5 antibody positive group (MDA5^+^ DM-ILD) and anti-MDA5 antibody negative group (MDA5^−^ DM-ILD) based on myositis-specific autoantibody test results. Demographic characteristics, lymphocyte subsets patterns and other clinical features were compared between the two groups. The association of lymphocyte subsets with 180-day mortality was investigated using survival analysis in MDA5^+^ DM-ILD.

**Results:**

Out of 253 eligible patients with DM-ILD, 59 patients were anti-MDA5^+^ and 194 were anti-MDA5^−^. Peripheral blood lymphocyte count, CD3^+^ count, percentage of CD3^+^, CD3^+^CD4^+^ count, and CD3^+^CD8^+^ count was lower in MDA5^+^ DM-ILD than in MDA5^−^ DM-ILD^−^ (all *P* < 0.001) as well as CD3^−^CD19^+^ count (*P* = 0.04). In MDA5^+^ DM-ILD, CD3^+^CD8^+^ count ≤ 49.22 cell/μL (HR = 3.81, 95%CI [1.20,12.14]) and CD3-CD19^+^ count ≤ 137.64 cell/μL (HR = 3.43, 95%CI [1.15,10.24]) were independent predictors of mortality. CD3^+^CD8^+^ count ≤ 31.38 cell/μL was associated with a higher mortality risk in all DM-ILD patients (HR = 8.6, 95%CI [2.12,31.44]) after adjusting for anti-MDA5 and other clinical characteristics.

**Conclusion:**

Significant lymphocytes decrease was observed in MDA5^+^ DM-ILD patients. CD3^+^CD8^+^ cell count was associated with worse prognosis in both MDA5^+^ DM-ILD and all DM-ILD patients.

**Supplementary Information:**

The online version contains supplementary material available at 10.1186/s12890-023-02706-y.

## Introduction

Dermatomyositis (DM) is an idiopathic inflammatory disease that causes muscle weakness and skin rashes. DM patients present different phenotypes and clinical courses that could be complicated with interstitial lung disease (ILD), which is associated with poor prognosis [1, 2]. A variety of myositis-specific antibodies (MSAs) had been identified for phenotyping of DM and early recognition of high risk patients, such as the most prevalent anti-Jo-1 (occurring in 9–24% of adult DM patients [3]), anti-melanoma differentiation-associated gene 5 (anti-MDA5, occurring from 15 to 20% in Asian DM patients [4]), and less common anti-PL-7, anti-EJ, anti-PL-12, anti-OJ, et al. [5, 6]. Anti-MDA5 has drawn increasing attention due to the high occurrence rate of ILD in anti-MDA5^+^ DM (MDA5^+^DM), among 50–70% [7, 8]. Anti-MDA5^+^ DM-ILD (MDA5^+^ DM-ILD) is associated with rapid progressive ILD, glucocorticoid resistance and often fatal outcomes [9–11].

The autoimmune mechanisms underlying MDA5^+^ DM-ILD are poorly understood [12–14]. Previous researches were mainly conducted in MDA5^+^ DM and showed that lymphocyte infiltration was involved in this pathogenesis [15]. Lymphocytes recruitment was found in the lung in MDA5^+^ DM patients and the circulatory lymphocytes were diminished, including the subsets T lymphocytes and B lymphocytes [16–18]. Lymphocytes targeted therapeutic has proved effective in the treatment of MDA5^+^ DM [19–21]. Further research of the immunological cellular characteristics in MDA5^+^ DM-ILD might help to understand the autoimmune mechanism underlying this high-risk subgroup and shed light to therapeutic methods.

Here we examined the immunological cellular characteristics in MDA5^+^ DM-ILD and explored possible prognostic factors.

## Method

### Patients

A total of 253 patients with DM who were diagnosed with ILD in the Department of Respiratory Medicine and the Department of Rheumatology and Immunology at Beijing Chao-Yang Hospital, Capital Medical University, Beijing Shijitan Hospital, Capital Medical University, and PLA Strategic Support Force Medical Center from January 1, 2016 to January 1, 2021 were included in this study. Demographic and medical records were obtained from the Electronic Medical Records (EMR) system. We recorded age, sex, smoking history, chronic disease, blood test results, lymphocyte subsets, MSAs spectrum, and survival status upon discharge. We conducted telephone follow-up 180 days after discharge.

### Inclusion and exclusion criteria

#### Inclusion criteria:

1. Aged between 18 and 80; 2. Compliance with the DM diagnostic criteria recommended by Bohan/Peter [22, 23] or Sontheimer's proposed CADM criteria (1999) [24]; 3. With ILD manifestations identified by chest HRCT; 4. With positive MSAs demonstrated by myositis antibody spectrum assay prior to treatment; 5. With complete test results of peripheral blood lymphocyte subsets present prior to treatment.

#### Exclusion criteria:

1. History of tumor or chronic lung disease; 2. Complicated by other connective tissue diseases, such as systemic sclerosis (SSc), Sjögren's syndrome (SS), rheumatoid arthritis (RA), and systemic lupus erythematosus (SLE); 3. Received systemic glucocorticoid and immunosuppressant treatment prior to hospitalization.

**Fig. 1:**
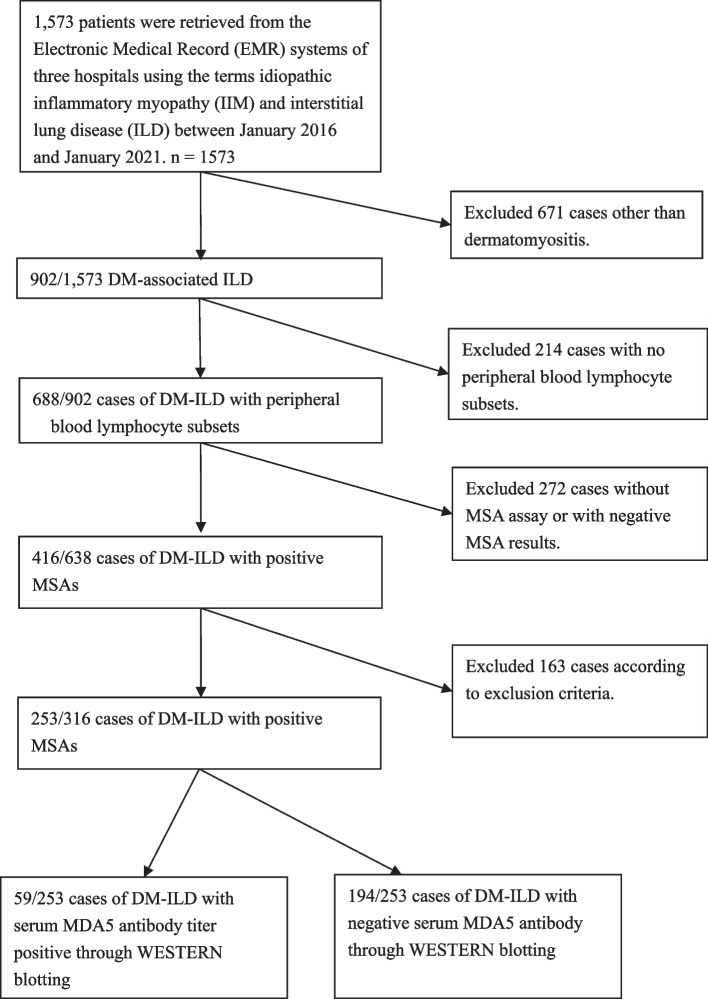
Sample selection profile. This flowchart shows how 253 DM-ILD patients were selected. The flowchart has six steps: Search EMR system for IIM with ILD. Exclude non-DM-ILD. Exclude the patients with no blood lymphocyte or MSA profile. Exclude the patients that met other exclusion criteria. Divide the remaining participants into MDA5^+^ DM-ILD and MDA5^−^ DM-ILD and enroll in the final cohort

All patients were anonymized. Based on EMR, 902 patients with DM-ILD were included from 1,573 patients with idiopathic inflammatory myopathy (IIM). After excluding 163 patients based on exclusion criteria, 253 patients had laboratory results for lymphocyte subsets and positive MSAs, including 59 patients with anti-MDA5 positive (MDA5^+^) and 194 patients with anti-MDA5 negative (MDA5^−^), were included in the analysis (Fig. 1).

### DM Serotyping (MSAs)

MSAs were assayed using WESTERN blotting. We defined MDA5^+^ DM-ILD as DM-ILD with positive anti-MDA5 antibody. Positivity for other MSAs was recorded.

### Imaging analysis

High-resolution computed tomography (HRCT) images were obtained from Picture Archiving and Communication System (PACS) in study centers. Patients with ground-glass opacity, cord-like and reticular fiber, and/or consolidation on chest HRCT were diagnosed as ILD. According to imaging and pathological characteristics, ILD was classified into usual interstitial pneumonia (UIP), nonspecific interstitial pneumonia (NSIP), organizing pneumonia (OP), diffuse alveolar damage (DAD), and mixed NSIP-OP. Mixed NSIP-OP is distinguished by a predominately basal fibrotic abnormality with superimposed OP [25]. The ILD diagnosis was confirmed through HRCT and weekly discussion by a multidisciplinary team (MDT) consisting of two pulmonary physicians specializing in interstitial lung disease, one rheumatologist, two radiologists, one pathologist, and one internist. If the MDT had a high level of confidence in radiological diagnosis (≥ 90%) and if HRCT radiological characteristics were typical, the MDT agreed that the type of ILD could be confirmed without a biopsy. In the case of atypical HRCT findings or objections to the pathological classification of ILD, the MDT engaged in deliberation. Rapidly progressive interstitial lung disease (RPILD) was defined as worsening radiological interstitial change with progressive dyspnea and hypoxemia within one month of the onset of respiratory symptoms [26].

### Lymphocyte subsets

Peripheral blood lymphocyte subsets were tested at the first visit with flow cytometry assay including counts and proportions of T lymphocytes (CD3^+^), helper/inducer T lymphocytes (CD3^+^CD4^+^ cells), suppressive/cytotoxic T lymphocytes (CD3^+^CD8^+^ cells), B lymphocytes (CD3^−^CD19^+^ cells), and NK cells (CD3^−^CD56^+^ cells).

### Statistical analysis

Categorical variables were summarized as frequency (proportion). Chi-squared test or the Fisher’s exact test were utilized to compare proportions between groups, as appropriate. Continuous variables were presented as mean (standard deviation) or median (25^th^ percentile, 75^th^ percentile). Normality of continuous variables were examined using Kolmogorov–Smirnov test. The Kolmogorov–Smirnov test was also used to compare continuous variables between groups as all variables rejected normality. The cutoff thresholds for continuous data were determined using the "*survminer*" R package and the maximum selection log-rank test. The Cox proportional hazard model was used to estimate the hazard ratios (HRs) and corresponding 95% Confidence Interval (95%CI) for all-cause mortality within 180 days, with the proportional hazard hypothesis investigated using Schoenfeld residuals. All analyses were performed using R Project for Statistical Computing (version 4.2.1). The two-sided *P* values < 0.05 were considered statistically significant.

## Results

### Baseline characteristics in MDA5^+^ DM-ILD and MDA5^−^ DM-ILD patients

From January 2016 to January 2021, 253 patients of DM-ILD were screened in the study, of whom 59 were MDA5^+^ DM-ILD and 194 were MDA5^−^ DM-ILD according to the inclusion criteria (Table 1). The median age of all patients was 55 years, with 68.4% being females. There was no significant difference in age and sex distribution between MDA5^+^ DM-ILD and MDA5^−^ DM-ILD patients (*P* = 0.106). During the 180-day follow-up, 30 (50.8%) deaths among MDA5^+^ patients and 15 (7.7%) were recorded among MDA5^−^ patients, which was significantly different (*P* < 0.001).

**Table 1:** Baseline characteristics in MDA5^+^ DM-ILD and MDA5^−^ DM-ILD patients

Baseline	MDA5^+^ DM-ILD cohort (*n* = 59)	MDA5^−^ DM-ILD cohort (*n* = 194)	*P* value
Age (years)	51 (46.5, 62.5)	57 (48.0, 64.0)	0.106
Female	37 (62.7%)	136 (70.1%)	0.285
Smoking history	11 (18.6%)	31 (16.0%)	0.63
180-day all-cause death	30 (50.8%)	15 (7.7%)	** < 0.001**

Table 1 compares the baseline characteristics of patients with DM-ILD who were positive or negative for MDA5 antibodies. The study included 59 MDA5^+^ DM-ILD patients and 194 MDA5^−^ DM-ILD patients. There were no significant differences between the two groups in terms of age, sex, and smoking history. However, the MDA5^+^ DM-ILD group had a significantly higher 180-day all-cause mortality rate than the MDA5.^−^ DM-ILD group (50.8% vs. 7.7%, *P* < 0.001)

*P* < 0.05 is in bold

**Fig. 2:**
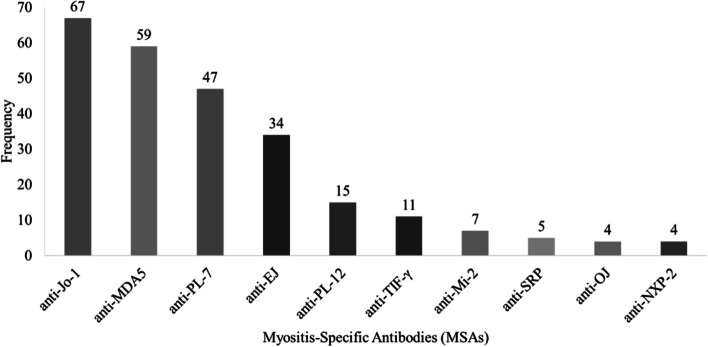
Distribution of MSAs among all DM-ILD patients (*N* = 253). Abbreviations: anti-Jo-1, anti-histidyl-tRNA synthetase; anti-MDA5, anti-melanoma differentiation-associated gene 5; anti-PL-7, anti-threonyl-tRNA synthetase; anti-EJ, anti-glycyl-tRNA synthetase; anti-PL-12, anti-alanyl-tRNA synthetase; anti-TIF1γ, anti-transcription intermediary factor-1γ; anti-Mi-2, anti-complex nucleosome remodeling histone deacetylase; anti-SRP, anti-signal recognition particle; anti-OJ, anti-isoleucyl-tRNA synthetase; anti-NXP-2, anti-nuclear matrix protein-2. Figure shows the distribution of MSAs in all DM-ILD patients. The most prevalent antibody was anti-Jo-1 (67 cases, 26.5%), followed by: anti-MDA5 (59 cases, 23.3%), anti-PL-7 (47 cases, 18.6%) and anti-EJ (34 cases, 13.4%). Other detected antibodies included anti-PL-12, anti-TIF1γ, anti-Mi-2, anti-SRP, anti-OJ and anti-NXP-2

Figure 2 depicts the distribution of MSAs from both cohorts. Among 253 patients with DM-ILD, 59 (23.3%) patients were anti-MDA5 positive. The majority of the 194 anti-MDA5^−^ patients were positive for anti-Jo-1 (*n* = 67), anti-PL-7 (*n* = 47), and anti-EJ (*n* = 34).

### MDA5^+^ DM-ILD exhibits more aggressive clinical traits

Lung imaging diagnosis results and relevant clinical characteristics are presented in Table 2. DAD was the most prevalent in MDA5^+^ DM-ILD patients, whereas NSIP was the most prevalent in MDA5^−^ DM-ILD patients. RPILD was observed in 49.2% of patients with MDA5^+^ DM-ILD (*n* = 29) compared to 5.2% in patients with MDA5^−^ DM-ILD (*n* = 10) (*P* < 0.001). Patients with MDA5^+^ DM-ILD had a longer average length of hospital stay (*P* = 0.005).

**Table 2:** Imaging study and clinical traits of patients in MDA5^+^ DM-ILD and MDA5^−^ DM-ILD cohorts

Characteristic	MDA5^+^ DM-ILD (n = 59)	MDA5^−^ DM-ILD (n = 194)	*P* value
**ILD pattern (Radiological and pathological diagnosis by MDT)**			
NSIP	20(33.9)	150(77.3)	** < 0.001**
OP	12(20.3)	21(10.8)	0.057
UIP	3(5.1)	5(2.6)	0.394
DAD	20(33.9)	1(0.5)	** < 0.001**
mixed NSIP-OP	4(6.8)	17(8.8)	0.791
**RPILD**	29(49.2)	10(5.2)	** < 0.001**
**length of hospital stay (d)**	14(8.00, 19.50)	12(8.00, 14.75)	**0.005**

Table 2 shows the differences in clinical, imaging, and pathological features between MDA5^+^ DM-ILD and MDA5^−^ DM-ILD. MDA5^+^ DM-ILD patients had a significantly lower rate of NSIP, and a significantly higher rate of DAD. MDA5^+^ DM-ILD patients also had longer hospital stays than MDA5^−^ DM-ILD patients

*P* < 0.05 are in bold

*Abbreviations*: *NSIP* nonspecific interstitial pneumonia, *OP* organizing pneumonia, *UIP* usual interstitial pneumonia, *DAD* diffuse alveolar damage, *RPILD* rapidly progressive interstitial lung disease

### MDA5^+^ DM-ILD Features more intensive lymphocyte depletion and activation

Comparison of peripheral blood lymphocyte subsets between MDA5^+^ DM-ILD and MDA5^−^ DM-ILD patients are presented in Table 3. The total number of lymphocytes in peripheral blood was significantly lower (*P* < 0.001) in MDA5^+^ patients than in MDA5^−^. In the MDA5^+^ DM-ILD patients, the percentage and count of CD3^+^ cells were significantly lower (both *P* < 0.001), as did the count of CD3^−^ CD19^+^ cells (*P* = 0.04). Analysis of subtypes of T-lymphocytes revealed a lower count of all subtypes (all *P* < 0.001). Sub-analysis of lymphocyte subsets in major types of MSAs DM-ILD (> 10% of total study population) showed a significantly lower total lymphocyte count, CD3^+^ cell count and CD3^+^CD4^+^ cell count (all *P* < 0.05) (Table 4).

**Table 3:** Peripheral blood lymphocyte subsets of patients in MDA5^+^ DM-ILD and MDA5^−^ DM-ILD cohorts

Lymphocyte subset	MDA5^+^ DM-ILD (*n* = 59)	MDA5^−^ DM-ILD (*n* = 194)	*P* value
blood lymphocyte count (× 10^9^/L)	0.66(0.46, 0.96)	1.075(0.78, 1.43)	** < 0.001**
CD3^+^ (%)	60.3(54.85, 64.85)	65.695(54.92, 75.58)	** < 0.001**
CD3^+^ (cell/μL)	402.97(248.18, 553.37)	659.5(445.75, 919.00)	** < 0.001**
CD3^+^CD4^+^ (%)	38.6(35.51, 44.85)	42.1(34.10, 48.58)	0.077
CD3^+^CD4^+^ (cell/μL)	255.51(171.55, 369.18)	433.5(275.25, 592.50)	** < 0.001**
CD3^+^CD8^+^ (%)	19.1(15.28, 22.99)	20.23(14.96, 26.78)	0.085
CD3^+^CD8^+^(cell/μL)	123(72.71, 200.48)	205.5(129.00, 316.50)	** < 0.001**
CD4^+^/CD8^+^	1.98(1.67, 2.75)	2.025(1.39, 2.85)	0.346
CD3^−^CD19^+^ (%)	18.5(14.15, 25.28)	17.38(9.85, 29.21)	**0.037**
CD3^−^CD19^+^ (cell/μL)	119.38(77.06, 191.95)	165.44(89.38, 313.84)	**0.04**
CD3^−^CD56^+^ (%)	12.7(9.80, 16.45)	9.3(6.00, 13.93)	** < 0.001**
CD3^−^CD56^+^ (cell/μL)	86.13(55.58, 134.70)	96.87(54.99, 155.80)	0.701

Table 3 compares the lymphocyte subsets of patients with DM-ILD according to their anti-MDA5 antibody status. The MDA5^+^ DM-ILD group had significantly lower counts of total lymphocytes, CD3^+^ cells, CD3^+^CD4^+^ cells, CD3^+^CD8^+^ cells, and CD3^−^CD19^+^ cells than the MDA5^−^ DM-ILD group. The MDA5^+^ DM-ILD group also had a lower percentage of CD3^+^ cells, but higher percentages of CD3^−^CD56^+^ cells and CD3^−^CD19^+^ cells than the MDA5^−^ DM-ILD group

*P* < 0.05 are in bold

**Table 4:** Peripheral blood lymphocyte subsets of patients with MDA5^+^ DM-ILD and other MSAs positive DM-ILD

Lymphocyte subset	MDA5^+^ DM-ILD (*n* = 59)	anti-Jo-1^+^ DM-ILD (*n* = 71)	anti-PL-7^+^ DM-ILD (*n* = 47)	anti-EJ^+^ DM-ILD (*n* = 34)
blood lymphocyte count (× 10^9^/L)	0.66(0.46, 0.96)	1.12(0.83, 1.53) ***	1.08(0.81, 1.44) ***	1(0.74, 1.32) **
CD3^+^ (%)	60.3(54.85, 64.85)	69.3(58.22, 77.98) ***	66.2(56.72, 74.75) **	58.9(51.85, 70.85)
CD3^+^ (cell/μL)	402.97(248.18, 553.37)	663(450.5, 980.5) ***	695(529.5, 914.74) ***	586(396, 917.1) *
CD3^+^CD4^+^ (%)	38.6(35.51, 44.85)	41.02(35.92, 50.18)	43.01(36.45, 48) *	42.86(34.15, 47.5)
CD3^+^CD4^+^ (cell/μL)	255.51(171.55, 369.18)	448(280.5, 651.5) ***	458(315,586) ***	387(243, 587.5) *
CD3^+^CD8^+^ (%)	19.1(15.28, 22.99)	21.7(16.4, 27.2) *	21.5(14.95, 27.99)	17.49(12.3, 23.44)
CD3^+^CD8^+^(cell/μL)	123(72.71, 200.48)	227(133, 334.5) ***	231(158.5, 301) ***	168(119, 272.5)
CD4^+^/CD8^+^	1.98(1.67, 2.75)	1.94(1.48, 2.71)	1.98(1.29, 2.95)	2.26(1.35, 3.28)
CD3^−^CD19^+^ (%)	18.5(14.15, 25.28)	13.61(9.7, 26.95) **	16.46(8.95, 26.86)	18.3(8.5, 30.7)
CD3^−^CD19^+^ (cell/μL)	119.38(77.06, 191.95)	152.4(75.77, 317.2)	176.3(86.91, 289.85)	166.95(58.31, 269.69)
CD3^−^CD56^+^ (%)	12.7(9.80, 16.45)	9(5.6, 12.9) ***	9(6.01, 13) ***	12.8(6.43, 16.6)
CD3^−^CD56^+^ (cell/μL)	86.13(55.58, 134.70)	79.52(44.8, 151.12)	105(61.2, 150.2)	113.12(72.74, 182.16)

Table 4 compares the lymphocyte subsets between MDA5^+^ DM-ILD, anti-Jo-1^+^ DM-ILD, anti-PL-7^+^ DM-ILD and anti-EJ^+^ DM-ILD. The MDA5^+^ DM-ILD group had significantly lower counts of total lymphocytes, CD3^+^ cells, CD3^+^CD4^+^ T cells compared to other groups of MSAs DM-ILD

^*^ *P* value < 0.05 compared with MDA5^+^ DM-ILD. ^**^ *P* value < 0.01 compared with MDA5^+^ DM-ILD. ^***^ *P* value < 0.001 compared with MDA5^+^ DM-ILD

### CD3^+^CD8^+^ Count and CD3^−^CD19^+^ count predict mortality in MDA5^+^ DM-ILD

**Fig. 3:**
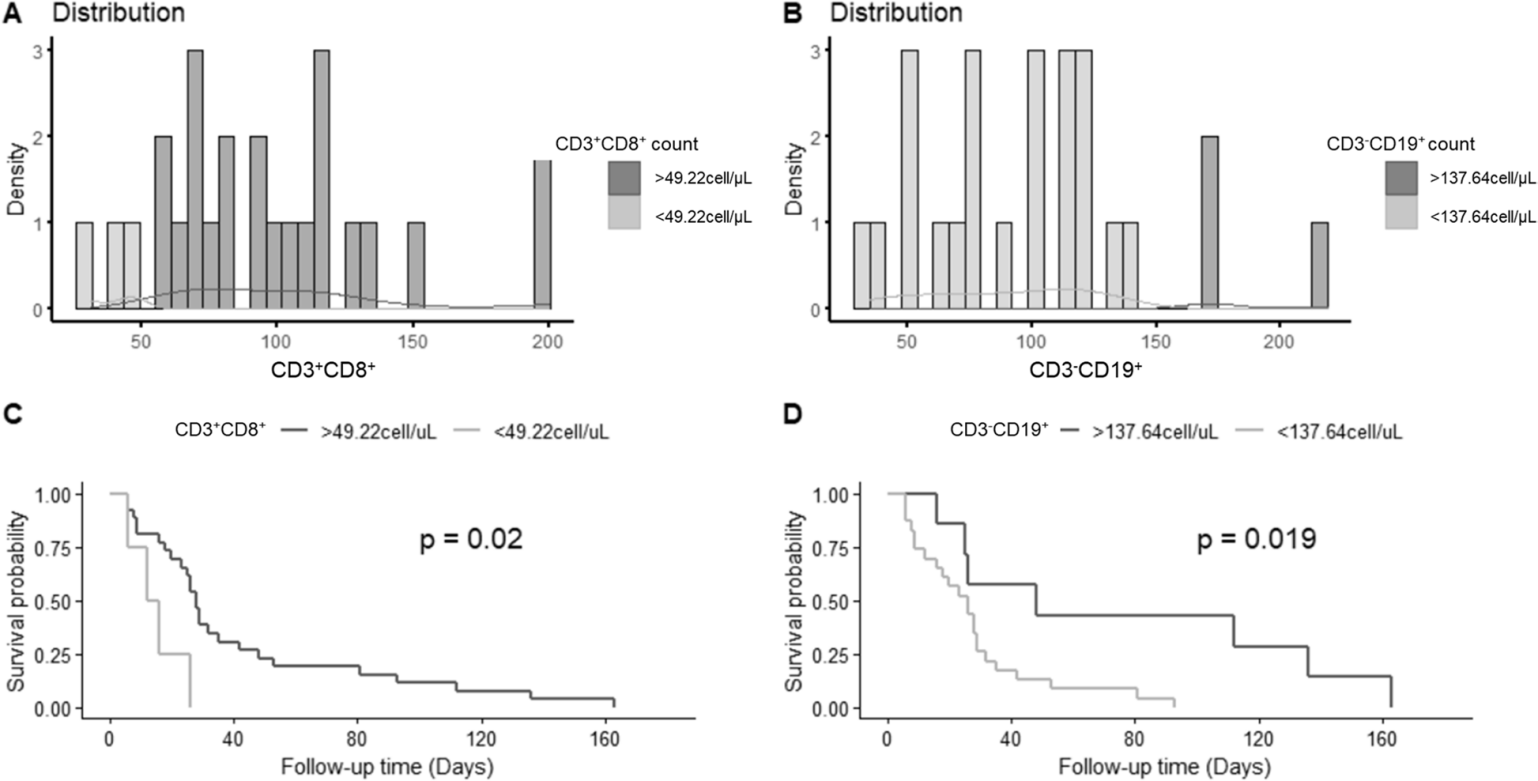
**A, B** Optimal cut-off values for CD3^+^CD8^+^ count and CD3^−^CD19^+^ count using the “*survminer*” R package. **C, D** The Kaplan–Meier survival curves displaying 180-day all-cause mortality, based on CD3^+^CD8^+^ count (with cut-off value of 49.22 cell/μL) and CD3^−^CD19^+^ count (with cut-off value of 137.64 cell/μL). Figure shows how the survival outcome of patients with MDA5^+^ DM-ILD is related to the counts of two types of lymphocytes: CD3^+^CD8^+^ T cells and CD3^−^CD19^+^ B cells. The cut-off value for CD3^+^CD8^+^ T cells is 49.22 cell/μL and the cut-off value for CD3^−^CD19^+^ B cells is 137.64 cell/μL. The histograms in panels A and C show the number of patients with recorded outcomes in each group. The Kaplan–Meier curves in panels B and D show the survival probability of each group over time. The log-rank tests show that both lymphocyte counts are significantly associated with survival outcome. Patients with higher CD3^+^CD8^+^ T cell count or higher CD3^−^CD19^+^ B cell count have a better prognosis than those with lower CD3^+^CD8^+^ T cell count or lower CD3^−^CD19^+^ B cell count. The *P*-values for the log-rank tests are 0.02 and 0.019, respectively

We compared lymphocyte subsets between survived and deceased patients in the MDA5^+^ DM-ILD cohort (Supplementary material 1). CD3^+^CD8^+^ count and CD3^+^CD19^+^ count was identified as significant predictors of mortality (Fig. 3A, B). Kaplan–Meier survival curves demonstrated statistically significant differences between groups (Fig. 3C, D, all *P* < 0.05). Table 5 displays the Cox regression hazard ratios (HR).

**Table 5:** Hazard ratio (HR) for prognostic factors in MDA5^+^ DM-ILD

Factor	Adjusted HR(95%CI)	*P* value
CD3^+^ cell count (< 233.12 cell/μL)	1.36(0.59,3.12)	0.47
CD3^+^ % (< 53.29%)	0.61(0.22,1.71)	0.35
CD3^+^CD4^+^ cell count (< 193.5 cell/μL)	1.41(0.63,3.13)	0.4
CD3^+^CD4^+^ % (< 38.7%)	0.44(0.18,1.04)	0.06
CD3^+^CD8^+^ cell count (< 49.22 cell/μL)	3.81(1.20,12.14)	**0.023**
CD3^+^CD8^+^ % (< 10.7%)	4.56(0.87,23.94)	0.073
CD3^+^CD4^+^ cell count/CD3^+^CD8^+^ cell count (< 2.99)	0.37(0.13,1.06)	0.06
CD3^−^CD19^+^ cell count (< 137.64 cell/μL)	3.43(1.15,10.24)	**0.027**
CD3^−^CD19^+^ % (< 27%)	3.45(0.84,14.19)	0.087
CD3^−^CD56^+^ cell count (< 67.62 cell/μL)	2.84(0.98,23.46)	0.052
CD3^−^CD56^+^ % (< 8.2%)	4.89(0.97,24.72)	0.055

Table 5 shows the results of the multivariate Cox regression analysis for MDA5^+^ DM-ILD survival. The analysis identified two independent prognostic factors that were significantly associated with increased mortality risk: CD3^+^CD8^+^ cell count lower than 49.22 cell/μL (HR = 3.81, 95% CI = 1.20-12.14, *P* = 0.023) and CD3^−^CD19^+^ cell count lower than 137.64 cell/μL (HR = 3.43, 95% CI = 1.15-10.24, *P* = 0.027)

*P* < 0.05 are in bold

### Anti-MDA5 positivity and CD3^+^CD8^+^ count independently predict mortality in all patients with DM-ILD

**Fig. 4:**
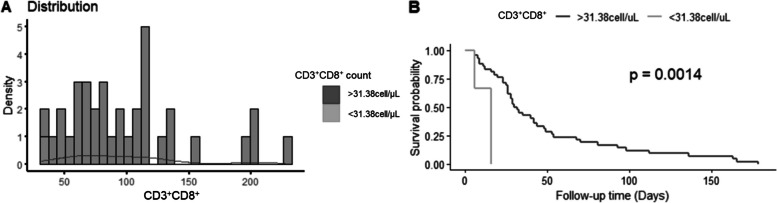
**A, B** Optimal cut-off value (31.38 cell/μL) and the Kaplan–Meier survival curves for CD3^+^CD8^+^ count using the “*survminer*” R package. Figure shows the association between CD3^+^CD8^+^ cell count and survival outcome in patients with DM-ILD. Figure 3A is a histogram that shows the frequency of patients with recorded outcomes in two groups: those with CD3^+^CD8^+^ cell count lower than 31.38 cell/μL (*n* = 2) and those with CD3^+^CD8^+^ cell count higher than 31.38 cell/μL (*n* = 43). Figure 3B is a Kaplan–Meier curve that shows the survival probability of the two groups over time. The log-rank test revealed a significant difference in survival between the two groups (*P* = 0.0014), with the higher CD3^+^CD8^+^ cell count group having a better prognosis than the lower CD3^+^CD8^+^ cell count group

Anti-MDA5 positivity was associated with higher mortality (HR = 2.08[1.64,13.22], *P* = 0.032) among all patients. CD3^+^CD8^+^  ≤ 31.38 cell/μL was associated with 180-day mortality (HR = 8.6[2.12,31.44], *P* = 0.002) after adjusting for sex, age, MDA5 status and RPILD (Table 6, Fig. 4A, B).

**Table 6:** Hazard ratio (HR) for prognostic factors in all DM-ILD

Factor	Adjusted HR(95%CI)	*P* value
Anti-MDA5 positive	2.08(1,64,13.22)	**0.032**
CD3^+^ cell count (< 420.29 cell/μL)	2.18(1,4.63)	0.052
CD3^+^ % (< 56.07%)	0.81(0.42,1.56)	0.53
CD3^+^CD4^+^ cell count (< 193.5 cell/μL)	1.68(0.9,3.15)	0.61
CD3^+^CD4^+^ % (< 36.15%)	0.85(0.41,1.76)	0.7
CD3^+^CD8^+^ cell count (< 31.38 cell/μL)	8.6(2.12,31.44)	**0.002**
CD3^+^CD8^+^ % (< 10.7%)	6.78(1.74,26.53)	0.006
CD3^+^CD4^+^ cell count/CD3^+^CD8^+^ cell count (< 2.97)	0.7(0.34,1.42)	0.32
CD3^−^CD19^+^ cell count (< 137.64 cell/μL)	2.02(0.97,4.24)	0.062
CD3^−^CD19^+^ % (< 27%)	2.3(0.91,5.1)	0.12
CD3^−^CD56^+^ cell count (< 40.32 cell/μL)	1.74(0.88,3.44)	0.11
CD3^−^CD56^+^ % (< 7.5%)	2.17(0.75,6.28)	0.15

Table 6 shows the results of the multivariate Cox regression analysis for DM-ILD survival. The analysis identified two independent prognostic factors that were significantly associated with increased mortality risk: anti-MDA5 antibody positivity (HR = 2.08, 95% CI = 1.64-13.22, *P* = 0.032) and CD3^+^CD8^+^ cell count lower than 31.38 cell/μL (HR = 8.6, 95% CI = 2.12-31.44, *P* = 0.002)

*P* < 0.05 are in bold

## Discussion

In this study, we examined 253 patients with DM-ILD, the largest number of participants to date. Specifically, Jo-1 was the most prevalent antibody, followed by MDA5 and PL-7, with MDA5 having a 23.3% positive rate (Fig. 2). The distribution of myositis antibodies in Asian populations was essentially consistent with previous reports [27]. Our study covered 59 patients with MDA5^+^ DM-ILD. This is the largest study to date in terms of the number of MDA5^+^ DM-ILD participants.

Compared to previous studies that focused on DM-ILD as a whole and with non-ILD DM patients as controls [18, 28, 29], we focused on MDA5^+^ DM-ILD by dividing DM-ILD into two groups based on MDA5 positivity in an attempt to obtain more pertinent clinical outcome indicators. By using MDA5^−^ DM-ILD as controls, it will be more conducive to elucidating the characteristics of lymphocyte subsets in the MDA5^+^ DM-ILD.

Data was collected prior to the treatment, therefore the influence of immunosuppressive drugs on lymphocyte subsets could be avoided. More DAD and RPILD were observed in the MDA5^+^ DM-ILD after patients’ enrollment, while more NSIP was observed in the control group. Follow up data showed a mortality rate of 50.8% in MDA5^+^ DM-ILD group and 7.7% in MDA5^−^ DM-ILD group during the 180-day length (Table 1), further confirmed the much worse prognosis of MDA5^+^ DM-ILD.

Compared to the MDA5^−^ DM-ILD group, the MDA5^+^ DM-ILD group exhibited significantly lower lymphocyte count, T lymphocyte count and B lymphocyte count. It was interesting to find that T and B lymphocytes decreased in an asynchronous fashion. T lymphocyte count was approximately 38.9% lower in MDA5^+^ DM-ILD than in MDA5^−^ DM-ILD, while B lymphocyte count was about 27.8% lower (Table 3). Data from Supplementary material also showed similar results when comparing death subjects to survival subjects. These results indicate that T lymphocytes might participate more in the pathogenesis. Further analysis on the lymphocyte subsets revealed that both CD3^+^CD4^+^ cell count and CD3^+^CD8^+^ cell count decreased, and their degrees of decrease were similar to that of total T lymphocytes. The mechanism underlying the lymphocytes decrease is largely unknown. Prior research has shown that the low T lymphocyte count in the peripheral blood of patients with DM can be attributed, in part, to the inhibited autophagy function of T cells, which promotes T lymphocyte apoptosis [30]. Furthermore, massive immune cell infiltration was identified in lung tissue of MDA5^+^ DM patients with ILD complications [31], it was hypothesized that activated lymphocytes in circulation were recruited to the target organs, such as lung [32]. The hypothesis was further supported by the increase of CD3^+^CD4^+^ count in alveolar lavage fluid of patients with DM [33]. Lymphocytes in peripheral blood metastasizing to the lungs where lymphatic vessels are abundant, participate in the local immune response and result in lymphopenia in peripheral blood. Similar results was found in B lymphocytes from tissues like muscle and lung biopsies [34, 35]. Future observations on the alterations of alveolar lavage fluid, lung biopsy tissue, and peripheral blood lymphocytes tracking in patients with MDA5^+^ DM-ILD and MDA5^−^ DM-ILD may provide additional support for the hypothesis.

Previous studies indicates that NK cells can release an excessive amount of IFN-γ, leading to pulmonary affection [36], and the total number of NK cells in myositis patients with pulmonary affection is lower than in those without pulmonary affection [37]. However, the count of peripheral blood NK cells did not differ significantly between the MDA5^+^ DM-ILD and MDA5^−^ DM-ILD groups in our study. The percentage of NK cells was statistically higher in the MDA5^+^ DM-ILD group, which might be explained by drastically decreased total number of lymphocytes. When comparing the death subjects to the survival subjects, the percentage of NK cells did not differ, but the cell count was significantly reduced. Further research is warranted to clarify whether NK cells play a role in determining the clinical course.

The regression analysis in MDA5^+^ DM-ILD patients showed that the poor prognosis was associated with low CD3^+^CD8^+^ and low CD3^+^CD19^+^ levels (HR were 3.81 and 3.43, respectively) (Table 5). When the analysis was performed in all DM-ILD, low CD3^+^CD8^+^ cell count was independent predictor of death (HR 8.6) even after adjusting for anti-MDA5 and other clinical characteristics, and the HR was much higher than that of anti-MDA5 (HR 2.08) (Table 6). So, a low CD3^+^CD8^+^ might be a better prognostic factor than anti-MDA5 and warrant further study.

### Limitation of the study

This study has several limitations. Firstly, the high prevalence of MDA5^+^ DM was unavoidable given that all of the clinical records collected for this retrospective study originated from grade A tertiary hospitals. Patient cohorts may represent a spectrum of more severe diseases due to referral bias. Secondly, all the participants were of East Asian descent, no other races were included. Thirdly, we did not continuously monitor post-treatment changes in lymphocyte subsets, which may have been associated with the treatment response. In the future, larger population-based multicenter studies will be required to obtain more accurate data.

## Conclusions

Patients with MDA5^+^ DM-ILD exhibited significant immune imbalance characterized primarily by diminished T and B lymphocytes. Peripheral blood lymphocyte subsets may serve as prognostic markers for MDA5^+^ DM-ILD and DM-ILD. Moreover, lower CD3^+^CD8^+^ is an independent risk factor for the prognosis of MDA5^+^ DM-ILD and DM-ILD, laying a foundation for further prognostic prediction and targeted therapy.

### Supplementary Information

**Additional file 1:** **Supplementary material 1.** Comparison of clinical data between survived and deceased patients in the MDA5^+^ DM-ILD cohort.

## Data Availability

The data that support the findings of this study are not openly available due to reasons of sensitivity and are available from the corresponding author upon reasonable request.
