# Supplementary material for: Characteristics and prognostic implications of peripheral blood lymphocyte subsets in patients with anti-MDA5 antibody positive dermatomyositis-interstitial lung disease
Source: BMC Pulm Med. 2023 Oct 28;23:411. doi: 10.1186/s12890-023-02706-y (PMC10612305; doi:10.1186/s12890-023-02706-y)
Supplement: Supplementary file 1 — Additional file 1: Supplementary material 1. Comparison of clinical data between survived and deceased patients in the MDA5+ DM-ILD cohort. [file 12890_2023_2706_MOESM1_ESM.docx]

**Supplementary Materials**

**Supplementary material 1 Comparison of clinical data between survived and deceased patients in the MDA5^+^ DM-ILD cohort**

| **Item** | **Survived（n = 29）** | **Deceased (n = 30)** | ***P* value** |
| --- | --- | --- | --- |
| **ILD pattern (Radiological and pathological diagnosis by MDT)** | | | |
| NSIP | 18(62.1%) | 2(6.7%) | **<0.001** |
| OP | 5(17.2%) | 7(23.3%) | 0.748 |
| UIP | 2(6.9%) | 1(3.3%) | 0.612 |
| DAD | 0(0.0%) | 20(66.7%) | **<0.001** |
| mixed NSIP-OP | 4(13.8%) | 0(0.0%) | 0.052 |
| **RPILD** | 0(0.0%) | 29(96.7%) | **<0.001** |
| blood lymphocyte count (×10^9^/L) | 0.87(0.68, 1.25) | 0.53(0.34, 0.66) | **<0.001** |
| **Peripheral Blood Lymphocyte Subsets** | | | |
| CD3^+^ (%) | 58.4(56.35, 65.30) | 60.7(53.33, 64.77) | 0.75 |
| CD3^+^ (cell/μL) | 518.42(402.97, 838.17) | 301.07(228.70, 415.34) | **<0.001** |
| CD3^+^CD4^+^ (%) | 38.38(35.20, 42.80) | 38.65(35.83, 45.53) | 0.88 |
| CD3^+^CD4^+^ (cell/μL) | 321.36(246.40, 451.00) | 200.97(145.34, 285.98) | **0.002** |
| CD3^+^CD8^+^ (%) | 20(15.60, 22.97) | 18.09(14.85, 22.68) | 0.808 |
| CD3^+^CD8^+^(cell/μL) | 166.97(123.00, 218.90) | 92.43(63.83, 123.09) | **<0.001** |
| CD4^+^/CD8^+^ | 1.95(1.64, 2.76) | 2.04(1.74, 2.72) | 0.861 |
| CD3^-^CD56^+^ (%) | 14.9(9.80, 16.50) | 12.55(9.80, 16.15) | 0.42 |
| CD3^-^CD56^+^ (cell/μL) | 115.5(69.70, 175.75) | 67.83(42.62, 92.58) | **0.007** |
| CD3^-^CD19^+^ (%) | 18.5(10.90, 25.26) | 18.52(15.28, 25.33) | 0.673 |
| CD3^-^CD19^+^ (cell/μL) | 171.76(88.80, 278.30) | 100.56(63.22, 130.30) | **0.017** |

*P* < 0.05 are in bold.

Abbreviations: NSIP, nonspecific interstitial pneumonia; OP, organizing pneumonia; UIP, usual interstitial pneumonia; DAD, diffuse alveolar damage; RPILD, rapidly progressive interstitial lung disease.
